# HIV prevalence and determinants of loss-to-follow-up in adolescents and young adults with tuberculosis in Cape Town

**DOI:** 10.1371/journal.pone.0210937

**Published:** 2019-02-05

**Authors:** Pancho Mulongeni, Sabine Hermans, Judy Caldwell, Linda-Gail Bekker, Robin Wood, Richard Kaplan

**Affiliations:** 1 The Desmond Tutu HIV Centre, Institute for Infectious Disease and Molecular Medicine, Faculty of Health Sciences, University of Cape Town, Cape Town, South Africa; 2 School of Public Health and Community Medicine, University of New South Wales, Sydney Australia; 3 Department of Global Health, Academic Medical Center, University of Amsterdam, Amsterdam Institute for Global Health and Development, Amsterdam, the Netherlands; 4 City Health, City of Cape Town, Cape Town, South Africa; University of KwaZulu-Natal, SOUTH AFRICA

## Abstract

TB remains a leading cause of mortality and morbidity in sub-Saharan Africa, due to the HIV epidemic. As TB treatment is lengthy, the completion of the full course of treatment may be especially challenging for young people. We therefore aimed to identify the extent of and reasons underlying loss to follow-up from TB treatment among young people in Cape Town. Accordingly, we reviewed the outcomes of young people treated for TB in Cape Town during 2009–2013, across three age groups: younger adolescents (10–14 years); older adolescents; (15–19 years) and young adults (20–24 years). We employed logistic regression analysis to identify risk factors for loss from TB care. 23,737 patients aged 10–24 were treated for drug sensitive TB over the study period. Of these, the HIV co-infection prevalence was 18.5% for younger adolescents, 12.9% for older adolescents and 33.1% for young adults. From age 16, HIV prevalence increased disproportionately among young women: by age 22, over 50% of women were TB/HIV co-infected compared to 14% of men. TB treatment success (cure plus completion) was 84.4%, while 1.7% of patients died, 9.5% were lost-to follow-up and 0.4% failed treatment. Being an older adolescent (aOR 1.75 [95% CI: 1.38–2.21]) or young adult (aOR: 1.96 [95% CI: 1.57–2.45]) increased the risk of loss-to-follow up, relative to being a younger adolescent. Further risk factors for loss from TB care were male gender (aOR: 1.33 [95% CI:1.20–1.46]), being a TB/HIV co-infected young person (aOR 1.74 [95% CI: 1.57–1.93]) and having had prior treatment for TB (aOR 3.17 [95% CI 2.87–3.51]). We identified risk factors for loss to follow-up and highlighted the need to focus on HIV prevention and retention in TB care among young people. TB care tailored to the needs of young people could improve patient retention, similar to improved outcomes reported by youth friendly HIV clinics.

## Introduction

In South Africa, tuberculosis (TB) remains one of the biggest causes of death during the productive years of life [1]. As human immunodeficiency virus-1 (HIV) infection became widespread in South Africa, the fraction of the population with immunosuppression expanded, resulting in a rise in TB disease [2]. In order to treat TB patients successfully, the health services need to successfully identify and treat TB/HIV co-infection. There has been some progress in this regard, with HIV testing among TB patients increasing from 59% to 93% over the course of 2009–14 [3]. However, successful TB treatment outcomes from 2012 to 2013 remained at roughly 77–78%, which is below the national target of 82% and far from the international targets of 85–90% [3].

While young people have been recognized as a priority population for the prevention of HIV [4], appreciation for the epidemiology of TB and the extent to which it overlaps with the HIV epidemic among young people has just begun to grow. Youthful populations face considerable challenges engaging with health services that are often tailored to adults and are insensitive to their developmental needs. Young people in particular, may have heightened difficulty remaining in TB care, because of adverse events related to treatment, as has been noted in high burden TB countries [5, 6]. Furthermore, as young people have not attained neurocognitive maturity they face—on average—greater difficulties in weighing the long-term consequences of their decisions compared to adults; an important consideration in understanding the reasons for which members of this population often discontinue lengthy treatment regimens [7]. Hence, a young person with TB may find it especially challenging to keep appointments with health providers and follow their recommendations [8].Youth friendly health services have been shown to increase retention in HIV treatment and Multi-Drug Resistant (MDR)-TB in sub-Saharan Africa [9, 10]. Such services may potentially improve drug sensitive TB treatment outcomes among young people in South Africa, but they are not widely available.

While earlier studies have focused on the prevalence of latent TB [1, 11, 12] and the incidence of active TB [13] in an adolescent cohort in South Africa, only three prior studies have addressed the outcomes of adolescents treated for TB in the country. Two of the studies enlisted only adolescents with multidrug resistant TB [6, 14] and are therefore of limited value in understanding how young people fair on treatment for drug sensitive TB. The most recent of the studies illustrated the TB epidemic among young people in the Western Cape Province, with a focus on HIV co-infection, notification rates and loss from TB care [15]. Yet this study only included new TB patients over a period of one year, overlooking young people who had prior TB and thus curtailing the usefulness of the findings in fully understanding youthful TB patient populations. No study has provided a risk factor analysis of the pathways through which young people on TB treatment in South Africa prematurely stop their treatment. Therefore, the question of which factors impact on retention in TB care–and the relationship thereof to HIV care for young people remains unanswered.

The aim of our study was to determine the burden of drug sensitive TB, across three different age groups, among young people in Cape Town and to assess TB treatment outcomes and the determinants thereof in this vulnerable group of patients. To this end, we estimated TB notification rates, prevalence of TB/HIV co-infection and determined TB treatment outcomes among younger adolescents, older adolescents and young adults. We also identified the risk factors for loss to follow-up from TB care.

## Methods

### Setting

Cape Town is the capital of the Western Cape Province of South Africa, and had an estimated population of approximately 3,851,163 in mid-2016 [16]. The city has a high prevalence of TB/HIV co-infection, with HIV prevalence among TB patients ranging between 44–50% during 2009–13 [17].

Patients with TB are treated at 101 primary care clinics across the city. Children, young people and adults with TB are treated in the same clinic, with routine TB care for ambulant patients provided by trained TB nurses. Medical officers provide supervision and support for the management of complicated cases [18].

Young people with TB received care in accordance with the South African national TB treatment guidelines [19]. Treatment duration was 6 months, with 8 months as the duration for patients with retreatment TB treated before 2012. After 2012, all drug sensitive new and retreatment TB patients received 6 months of TB treatment unless there was a clinical indication to extend the treatment duration [20].

### Design and study population

Retrospective cohort study of all young people (aged between 10 and 24 years), treated for drug susceptible TB in Cape Town primary care TB clinics between 2009 and 2013.

Data were obtained from the electronic TB register of City Health, City of Cape Town (ETR.net). Missing values were not replaced. Patients with drug-resistant tuberculosis (DR-TB) were also not included in the analysis, as they are recorded in a separate database.

As the ETR.net database contains no identifying information, we were unable to identify instances of one patient receiving their TB treatment course across more than one facility. For this reason, all patients who transferred into a facility were removed from the database. This avoided double counting of patients who begin their TB treatment course at one facility and then continue treatment at another facility.

### Data sources and measurements

For all patients, age, sex, date of treatment initiation, completion or last date of contact with health facility, type of TB (new or retreatment), anatomical site (pulmonary tuberculosis (PTB) or extrapulmonary tuberculosis (EPTB)), HIV status and TB treatment outcomes were extracted from the database. For HIV positive patients, CD4+ T-cell (CD4) count and antiretroviral therapy (ART) status at start of TB treatment were also extracted. Annual population estimates for Cape Town were obtained from mid-year estimates by Statistics South Africa [16].

#### Definitions

HIV status was confirmed according to an algorithm that accounted for HIV test results, ART status and CD4 count, as described previously [21]. A patient was defined to be HIV positive in three ways: either the patient had an HIV positive result recorded in the database or the patient was on ART or co-trimoxazole prophylaxis,or had a CD4 count measurement recorded upon entry into TB care. HIV negative TB patients were those who had an HIV negative test recorded in the database. All other patients were considered to have an unknown HIV status.

We defined three age categories, younger adolescents (10–14 years), older adolescents (15–19 years) and young adults (20–24 years), according to a framework proposed in a recent review of the causes of mortality and morbidity in young people [4].

The treatment outcomes of cure, completion, failure and transfer out followed the WHO definitions [22]. Treatment success was defined as the sum of cure and completion. Death comprised all-cause mortality during TB care.

Patients who interrupted TB treatment (failed to collect medications) for a period of at least 2 months were considered to have become Lost To Follow-Up. The date of loss to follow-up was defined as the last recorded date TB medications were collected. Facilities undertook efforts to trace these patients. However, in spite of efforts to recall, those patients who failed to return to care (after a period of at least 2 months) were deemed Lost to Follow-Up

In the event previous TB patients returned to TB care, after an absence of at least two months regardless of the first outcome, they were classified as retreatment cases on TB treatment re-initiation.

#### Data analysis

Data analysis was performed in Stata (Stata Corp 2013, Stata Statistical Software: Release 13. College Station, TX: StataCorp LP), R (The R Foundation for Statistical Computing, Version 3.2.3), using the RStudio interface (RStudio,Inc. Version 0.99.896) and Microsoft Excel 2013 (Microsoft Corp 2013).

#### Descriptive statistics

To estimate TB notification rates among young people in Cape Town, the annual number of TB patients per age group (from ETR.net) was divided by the age-specific population estimates for 2009–13 [16]. Descriptive statistics of HIV prevalence, ART usage and TB treatment outcomes were calculated by age category.

#### Inferential statistics

To assess the possibility of an association between age group and EPTB in younger adolescents relative to either older adolescents or young adults, Pearson’s Chi Squared Test statistic was computed. The Cochrane-Armitage Test was used to assess the significance of trends in the prevalence of retreatment TB, among all TB patients, in addition to the outcomes of death and loss to follow-up, across the three age categories.

#### Survival analysis

Events were defined as having loss to follow-up as an outcome, all other outcomes were defined as right-censored. Kaplan-Meier curves were drawn to illustrate differences in risk of loss to follow-up by age group. The log-rank test was used to test for heterogeneity in risk of loss to follow-up across age groups. Testing of the assumption of proportional hazards, by using scaled Schoenfeld residuals, was undertaken to assess the appropriateness of Cox-proportional hazards regression for modelling. Should there be sufficient evidence to reject the assumption of proportional hazards, the Cox-proportional hazards regression is rejected as a method of analysis. Logistic regression is then used as the appropriate technique to identify risk factor for loss to follow-up.

#### Multivariable modelling

Logistic regression analysis was performed to identify risk factors for loss from TB care for the entire cohort then separately for those adolescents living with HIV. In addition to all the co-variates in the model for the entire cohort, the model among only HIV positive adolescents included CD4+ count category and ART status at the start of TB treatment. Univariable models (with a threshold of p<0.05 for significance of effect size) were used to identify risk factors for inclusion in the multivariable model using backward selection.

#### Ethics

Approval for use of patient data was granted by City Health Directorate, City of Cape Town. The initial data collection was for a routine monitoring and evaluation process of the South African National TB Control Program rather than for an academic study. As such, patients were not asked to give informed consent for use of their clinical records. Patient confidentiality was assured by removing all identifiers from the dataset prior to use by investigators. The study was conducted in compliance with Good Clinical Practice guidelines and the principles of the Declaration of Helsinki. Ethical approval for this study was obtained by the Human Research Ethics Committee at the Faculty of Health Sciences, University of Cape Town, Cape Town, South Africa.

## Results

### Patient characteristics

During the course of 2009–2013, a total of 23,737 adolescent and young adult patients (aged 10–24 years) were registered for treatment of drug sensitive TB across 101 primary care health facilities in Cape Town. Of these 8.5% were younger adolescents (aged 10–14 years), 29.7% were older adolescents (aged 15–19 years) while young adults (20–24 years) constituted the bulk of the patients (61.8%). Across age categories, females comprised 54.6% of the TB patients. Demographic and clinical characteristics of adolescent TB patients by age groups are shown in Table 1.

**Table 1:** Baseline characteristics of adolescents and young adults treated for TB in Cape Town during 2009–13.

Age category (years)	10–14	15–19	20–24	Total
	N (%)	N (%)	N (%)	N (%)
**All patients**	2017 (100%)	7046 (100%)	14674 (100%)	23737 (100%)
HIV status				
HIV Negative	1536 (76.2%)	5889 (83.6%)	9393 (64.0%)	16818 (70.9%)
HIV Positive	373 (18.5%)	909(12.9%)	4855 (33.1%)	6137(25.9%)
Unknown status	108 (5.4%)	248 (3.5%)	426 (2.9%)	782 (3.3%)
Gender				
Female	1131 (56.1%)	3873 (55.0%)	7957 (54.2%)	12961 (54.6%)
Male	886 (43.9%)	3173 (45.0%)	6717 (45.8%)	10776 (45.4%)
Classification				
PTB	1646 (81.6%)	6197 (88.0%)	12540 (85.5%)	20383 (85.9%)
EPTB	371 (18.4%)	849 (12.0%)	2134 (14.5%)	3354 (14.1%)
Category				
New TB	1914 (94.9%)	6323 (89.7%)	12146 (82.8%)	20383 (85.9%)
Retreatment TB	103 (5.1%)	723 (10.3%)	2528 (17.2%)	3354 (14.1%)
HIV positive patients*				
CD4 count <100	68(18.2%)	156 (17.2%)	1267 (26.1%)	1491(24.3%)
CD4 count: 100–199	51 (13.7%)	208 (22.9%)	1050 (21.6%)	1309 (21.3%)
CD4 count: 200–349	81 (21.7%)	225 (24.8)	1169 (24.1)	1475(24.0%)
CD4 count: 350–499	55 (14.7%)	151 (16.6%)	684 (14.1%)	890 (14.5%)
CD4 count > = 500	76 (20.4%)	126 (13.9%)	499 (10.3%)	701(11.4%)
Unknown CD4 count	42 (11.3%)	43 (4.7%)	186 (3.8%)	271 (4.4%)
Median CD4 count (IQR)	279 (131–486)	239 (128–391)	201 (92–352)	210 (98–365)
ART naïve at start of TB Rx	255 (68.1%)	770 (84.7%)	4082 (84.1%)	5107 (83.2%)
On ART at start of TB Rx	118 (31.6%)	139 (15.3%)	770 (15.9%)	1027 (16.7%)
Unknown ART status	0	0	3 (0.1%)	3 (0%)

*The CD4 count and ART status at start of TB Rx refer to the 6137 HIV positive patients

ART, Antiretroviral Therapy; EPTB, Extrapulmonary Tuberculosis; PTB, Pulmonary Tuberculosis; Rx, treatment course.

Most young people presented with PTB across the age categories, with a higher fraction of EPTB patients in younger adolescents (18.4%) than in either of the older age categories (12.0% in older adolescents and 14.5% in young adults, p<0.001). Most adolescents had new TB, with the fraction of retreatment TB increasing steadily across the three age categories (5.1% in 10–14 year olds, 10.3% in 15–19 year olds and 14.3% in 20–24 year olds, p<0.001).

### Case Notification Rates (CNR)

**Fig 1:**
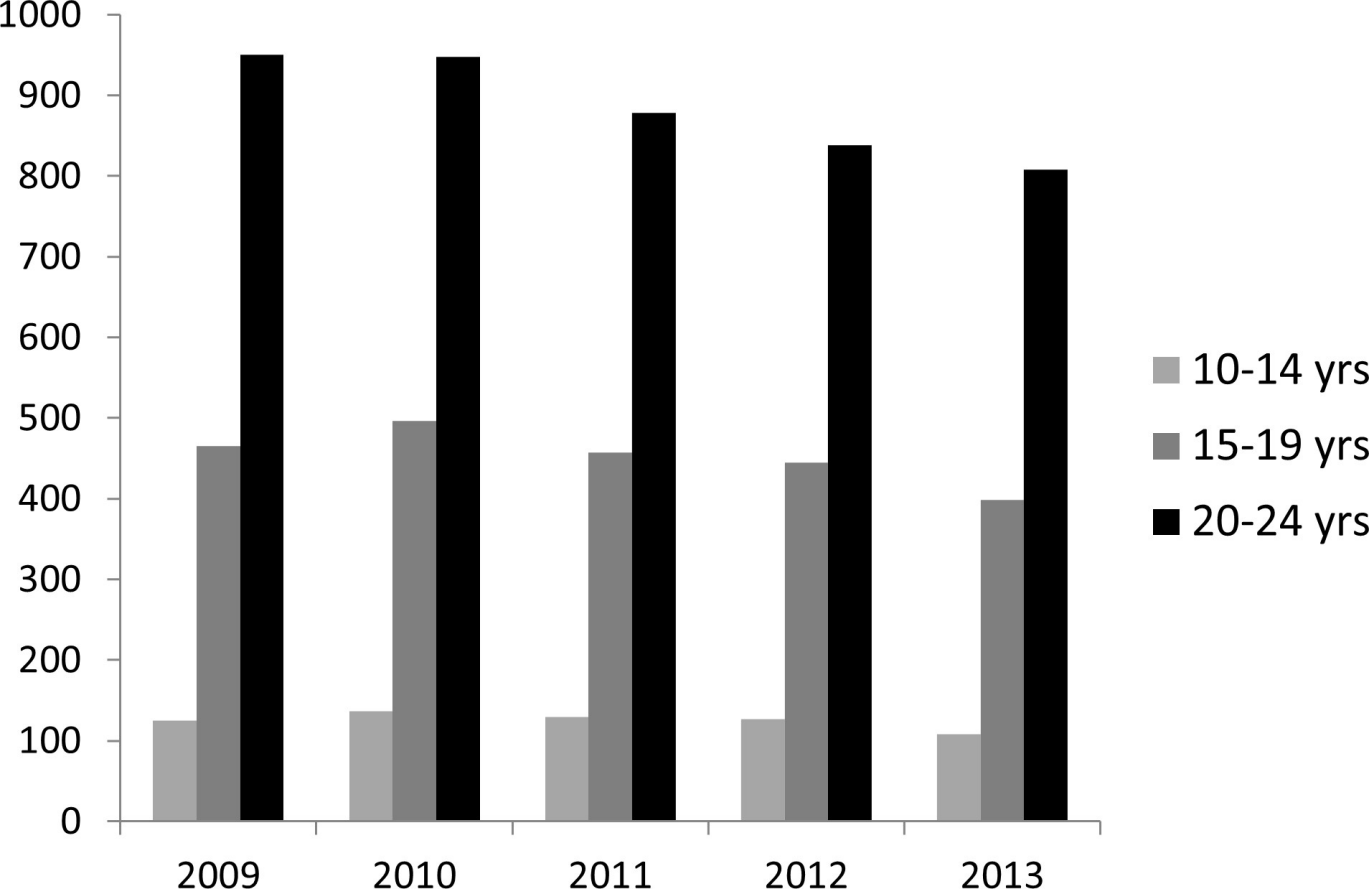
Trends in case notification rates per 100 000 population by age group, in Cape Town.

The estimated CNR among adolescents over the period 2009 to 2013 are presented in Fig 1. Older age categories had higher rates of TB. Overall, CNR declined across all three age categories over the study period.

### TB/HIV co-infection

**Fig 2:**
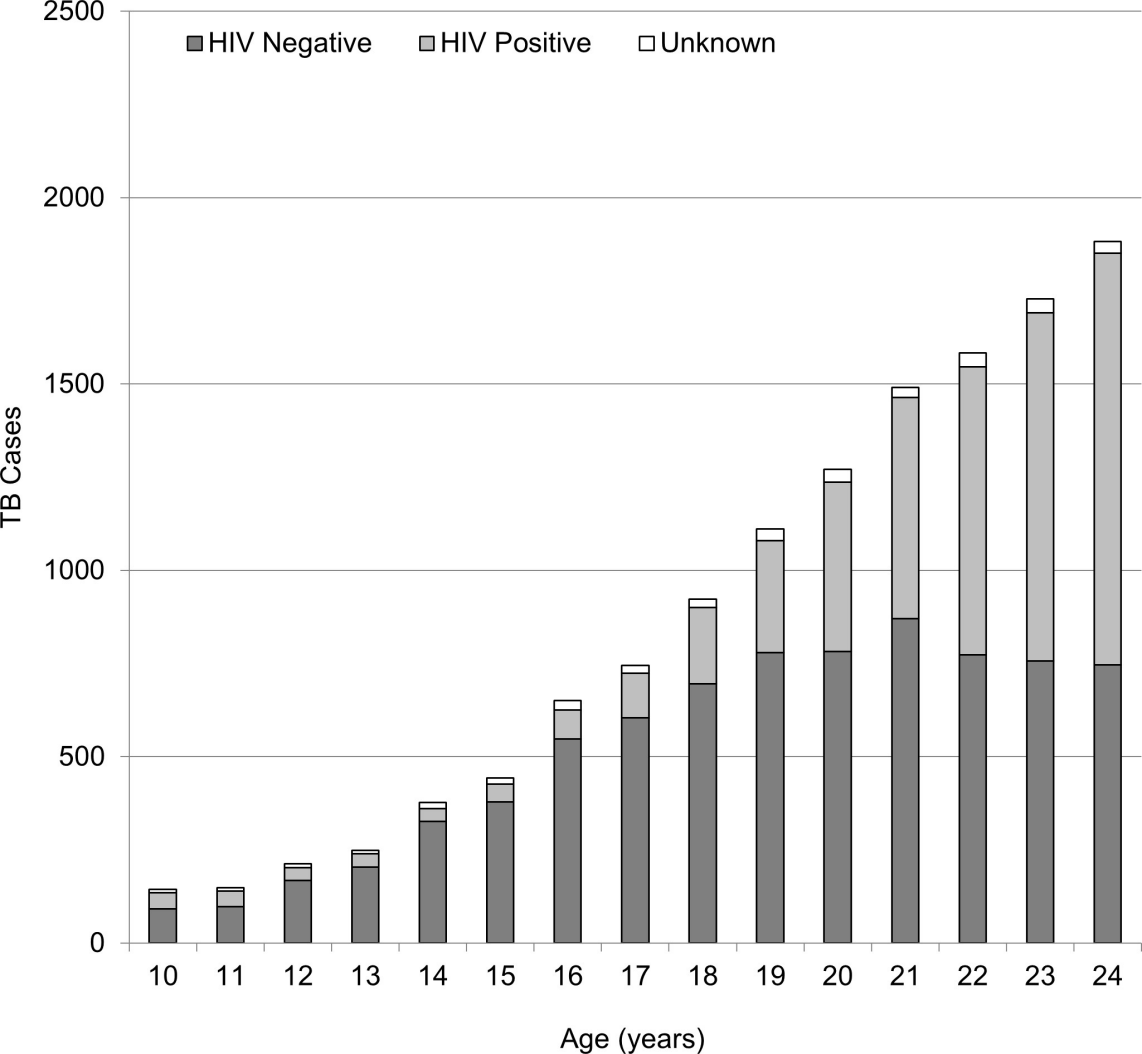
Prevalence of TB/HIV co-infection, across age categories, for female adolescents and young women treated for TB in Cape Town during 2009–13.

**Fig 3:**
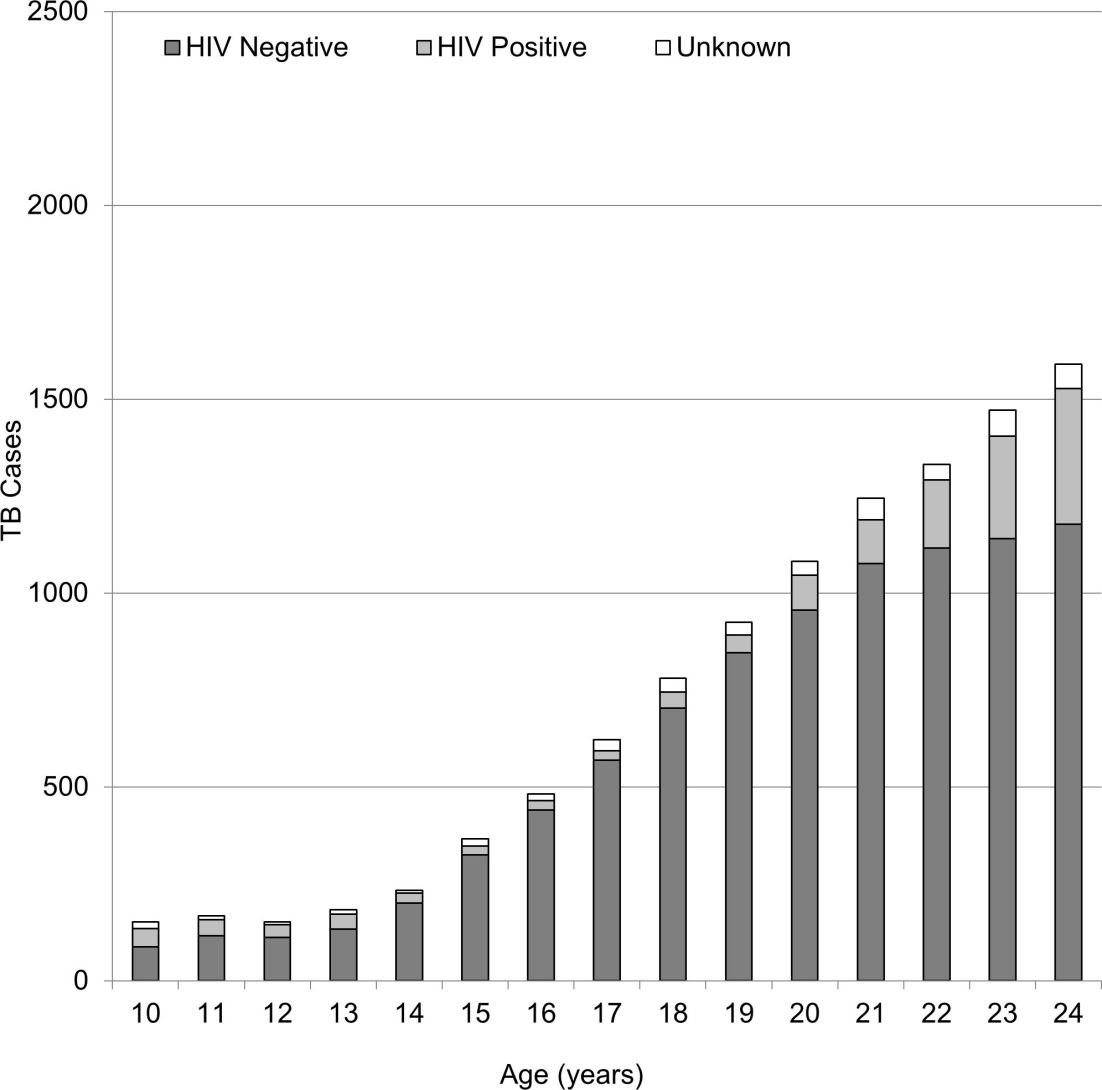
Prevalence of TB/HIV co-infection, across age categories, for male adolescents and young men treated for TB in Cape Town during 2009–13.

The prevalence of TB/HIV co-infection was highest among young adults (33.1%), followed by younger adolescents (18.5%) and older adolescents (12.9%) (Table 1). When disaggregating these age groups into individual age year bands, the rise of HIV prevalence with age was apparent (Figs 2 and 3). Starting from the age of 16, there was a steady increase in HIV prevalence in young women with TB. By the age of 22 more than 50% were HIV co-infected (Fig 2), in contrast to 14% among young men with TB (Fig 3). The median CD4 count among HIV co-infected adolescents at start of TB treatment was highest in younger adolescents and was markedly lower in the older age groups.

**Fig 4:**
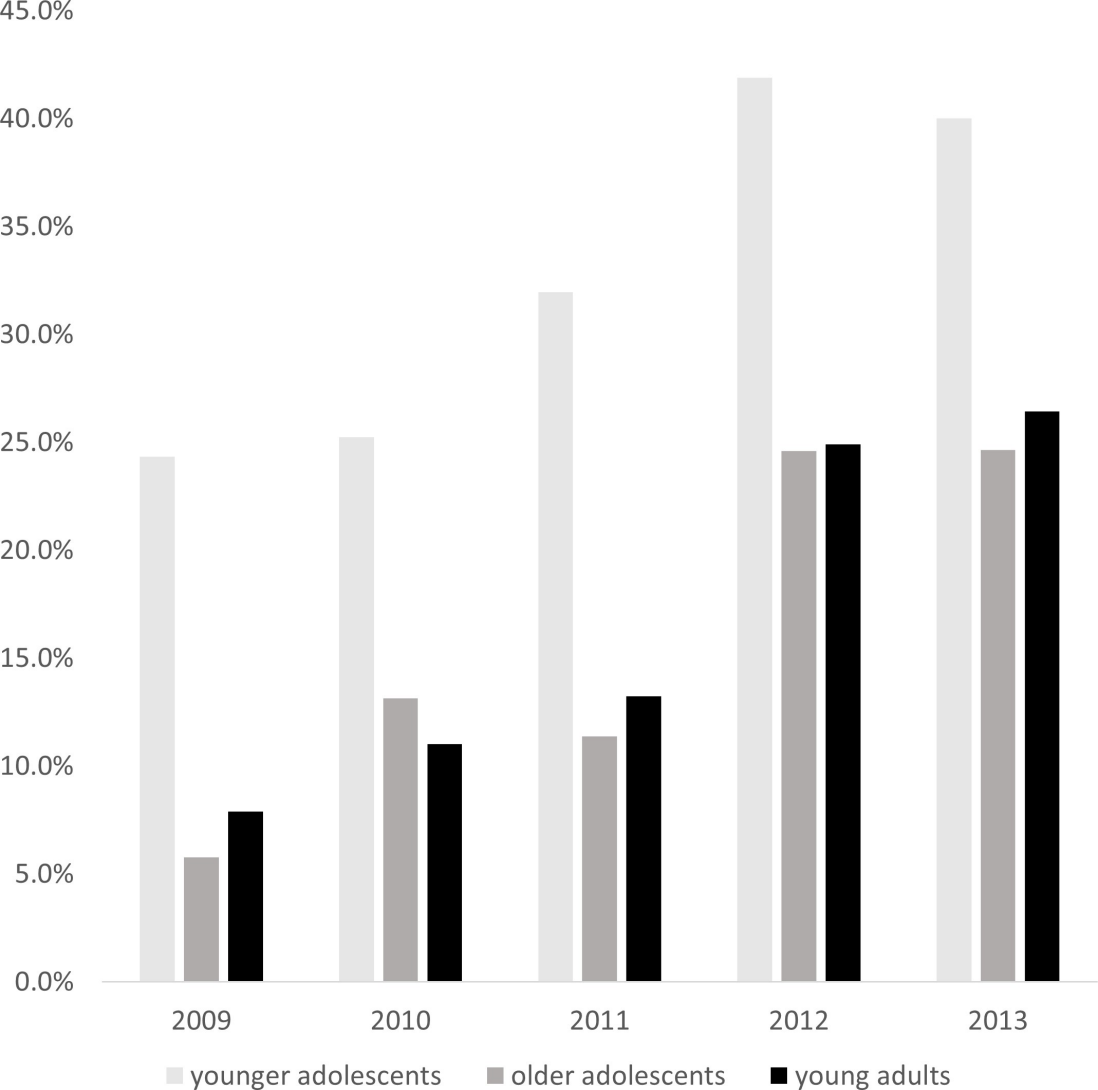
Proportion of younger adolescents, older adolescents and young adults with TB/HIV coinfection on ART at start of TB treatment per calendar year.

Furthermore, the proportion of TB/HIV co-infected young people who were on ART at the start of TB treatment was largest in the younger adolescents (31.6%), roughly double that of older adolescents and young adults on ART on entering TB care (Table 1). Over the course of the study period, the proportion of TB/HIV co-infected young people who were already enrolled in ART care at the time of enrolment in TB care increased across all age groups despite fluctuations in this fraction from one year to the next (Fig 4). Overall, among all HIV positive adolescents and young adults, the increase was from 8% on ART at time of enrolment in 2009 to 27% in 2013.

### TB treatment outcomes

The TB treatment outcomes are shown in Table 2. TB treatment success rates ranged from 82.0% in young adults to 92.0% in younger adolescents. High success rates in the younger age categories were commensurate with lower rates of loss to follow-up, with 1 in 20 younger adolescents becoming lost to follow-up on treatment compared to 1 in 9 young adults (p<0.001). Mortality also increased with age group, increasing from 0.5% in younger adolescents to 2.2% in young adults (p<0.001).

**Table 2:** TB treatment outcomes of adolescent and young adult TB patients.

	10–14 years	15–19 years	20–24 years	Total
Total, n	2017 (100%)	7046 (100%)	14674 (100%)	23737 (100%)
Cure plus completion, n (%)	1858 (92.0%)	6143 (87.2%)	12033 (82.0%)	20034 (84.4%)
Loss to follow-up, n (%)	90 (4.5%)	570 (8.1%)	1596 (10.9%)	2256 (9.5%)
Died, n (%)	10 (0.5%)	67 (1%)	328 (2.2%)	405 (1.7%)
Failed, n (%)	6 (0.3%)	26 (0.4%)	71 (0.5%)	103 (0.4%)
Moved or transferred, n (%)	32 (1.6%)	140 (2.0%)	445 (3.0%)	617 (2.6%)
Not evaluated, n (%)	21 (1%)	100 (1.4%)	201 (1.4%)	322 (1.4%)

n, number.

### Survival analysis

**Fig 5:**
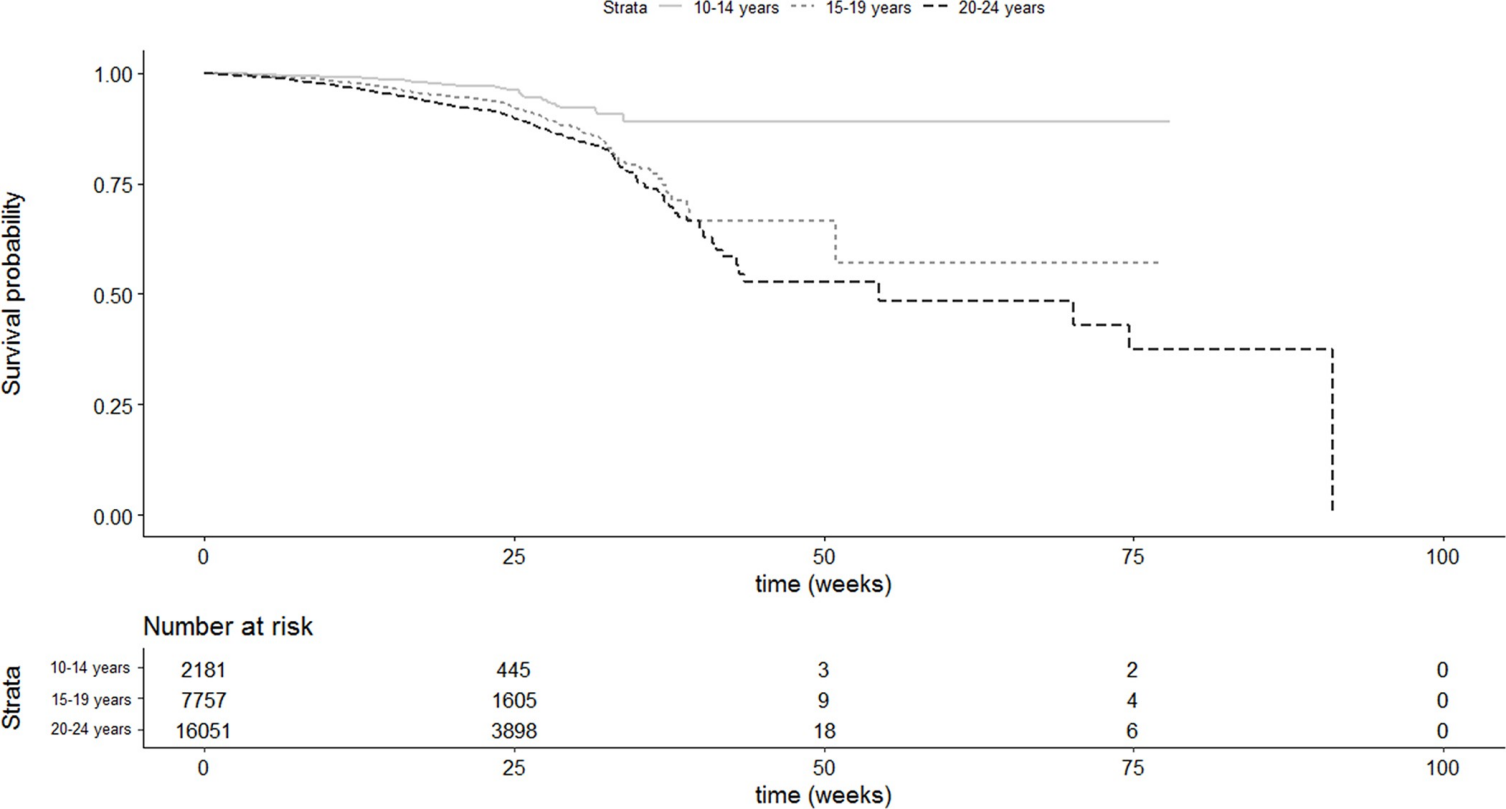
Kaplan Meier survival curve for loss to follow-up among young people in TB care by age group.

The Kaplan-Meier curves present differences in retention in care during the course of TB treatment among young people according to age group. Young adults were more likely to become lost to follow-up relative to older and younger adolescents (Fig 5) (p<0.001).

### Associations with loss to follow-up

Multivariable logistic regression of risk factors for loss to follow-up during TB care of young people was performed and is shown for the entire cohort, in Table 3, and for TB/HIV co-infected patients, in Table 4.

**Table 3:** Associations with loss to follow-up from TB treatment for the entire cohort.

Covariates	Unadjusted OR (95% CI)	P-value	Adjusted OR (95% CI)	P-value
*Age*				
10–14 year olds	1		1	
15–19 year olds	1.88 (1.5–2.37)	<0.001	1.75 (1.38–2.21)	<0.001
20–24 year olds	2.61 (2.10–3.25)	<0.001	1.96 (1.57–2.45)	<0.001
*Gender*				
Female	1		1	
Male	1.50 (1.05–1.25)	<0.001	1.33 (1.20–1.46)	<0.001
HIV status				
Negative	1		1	
Positive	1.75 (1.60–1.93)	<0.001	1.74 (1.57–1.93)	<0.001
*TB classification*				
Pulmonary	1		1	
EPTB	0.72 (0.62–0.82)	<0.001	0.80 (0.69–0.93)	0.003
*Past history of TB*				
New TB	1		1	
Retreatment of TB	3.53 (3.20–3.89)	<0.001	3.17 (2.87–3.51)	<0.001

EPTB, Extrapulmonary Tuberculosis; OR, odds ratio; PTB, Pulmonary Tuberculosis

**Table 4:** Associations with loss to follow-up from TB treatment for the HIV positive cohort.

Covariates	Unadjusted OR (95% CI)	P-value	Adjusted OR (95% CI)	P-value
*Age*				
10–14 year olds	1		1	
15–19 year olds	1.95 (1.26–3.01)	0.003	1.85 (1.17–2.91)	0.008
20–24 year olds	2.05 (1.38–3.06)	<0.001	1.88 (1.23–2.85)	0.003
*Gender*				
Female	1		1	
Male	1.19 (1.00–1.42)	0.05	1.27 (1.06–1.52)	0.01
CD4 counts*				
<100	1		1	
100–199	1.07 (0.86–1.34)	0.54	1.13 (0.91–1.43)	0.27
200–349	1.22 (0.99–1.51)	0.07	1.30 (1.05–1.61)	0.02
350–499	0.99 (0.77–1.28)	0.93	1.04 (0.81–1.35)	0.74
> = 500	1.28 (0.98–1.65)	0.07	1.34 (1.03–1.75)	0.03
ART naïve at start of TB treatment	1		1	
on ART at start of TB treatment	0.89 (0.73–1.09)	0.27	0.89 (0.72–1.75)	0.27
Pulmonary TB	1		1	
EPTB	0.86 (0.71–1.04)	0.11	0.97 (0.80–1.18)	0.80
*Past history of TB*				
New TB	1		1	
Retreatment of TB	2.24 (1.90–2.64)	<0.001	2.23 (1.88–2.65)	<0.001

*Refers to CD4 count at time of enrolment in TB care

ART, Antiretroviral Therapy; EPTB, Extrapulmonary Tuberculosis; OR, odds ratio

In the entire cohort, there was evidence to suggest an association between several risk factors included in the model and loss to follow-up (S1 Table**)**. Having had a previous TB episode, relative to a new episode, had the largest effect size (a roughly threefold increase in relative odds) of all factors included. Young adults had a nearly two-fold increase in relative odds of loss to follow-up compared to younger adolescents. Being HIV-positive, compared to HIV-negative, and being an older adolescent, relative to being a young adolescent, were each associated with an increase in relative odds of loss to follow-up by roughly 75%. Male patients had a roughly one-third increased relative odds of loss to follow-up compared to female patients. In contrast to these trends, patients with EPTB had a one-fifth lower relative odds of loss to follow-up compared to patients with PTB (Table 3).

In the TB/HIV co-infected cohort, evidence also suggested a similar pattern of loss-to-follow up to that of the entire cohort (S2 Table). Importantly, we found no significant association between being on ART at start of TB treatment and odds of loss to follow-up in the TB/HIV co-infected cohort (aOR 0.89 (95% CI: 0.72–1.75) (Table 4).

## Discussion

We have presented data on the burden of TB disease, TB treatment outcomes and the degree of TB/HIV co-infection among young people in Cape Town, during 2009–2013. In addition, our study elucidates risk factors for loss to follow-up from TB care among this vulnerable population. Outside South Africa there are few studies from resource-limited settings on TB treatment outcomes among young people. Studies from Brazil and India have assessed clinical features of young people with TB [23–25], with little reference to treatment outcomes. Our study follows on that of young people with TB in the Western Cape to provide a population-level analysis of TB treatment outcomes and TB/HIV co-infection among adolescents with active TB disease [15]. However, unlike the former study, we conducted an exhaustive analysis to illustrate both sex and age distribution of TB/HIV coinfection, as well as elucidating possible pathways through which adolescents and young adults become vulnerable to loss from TB care.

In Cape Town, the overall annual estimates of TB rates per 100 000 population lie between 850 in 2009 and 700 in 2013 [17]. These are higher than our estimates of the TB rates among younger and older adolescents. However, the estimates of the TB notification rates among young adults (aged 20–24 years) from this study are higher than the overall Cape Town annual estimates and greater than those reported for entire Western Cape, of which Cape Town is the largest city. These results indicate that young adults in Cape Town have a higher burden of TB than the average burden for the entire province. However, differences in calculation of notification rates between our study and that of the entire province render comparisons between notifications difficult, especially since we included retreatment patients. In summary, these results identify that young adults are a vulnerable group for TB disease in Cape Town.

Earlier studies on treatment outcomes for young people in TB care have failed to identify a consistent trend between age and loss to follow up. Similar to our study, Lotfian et al. reported on outcomes of adolescent TB patients in Iran and did not find differences in loss- to-follow-up between younger and older adolescents. However the small sample size of that study precluded investigators from performing the statistical modelling required to identify independent risk factors for loss-to-follow up [26]. In addition, a study in India found no loss to follow-up in their sample of young people in TB care, but here too the small sample size of the study, in comparison to the large adolescent population at risk of TB disease in that country, means the findings are unlikely to be generalizable[27]. However a recent study from Botswana that employed a substantial sample of young people in TB care, found higher loss to follow-up among adolescents (10–19 years of age), compared to young people (20–24 years of age) and adults (older than 24 years) [28]. In contrast, Snow et al., illustrated that older adolescents (15–19 years of age) and young adults (20–24 years of age) had markedly higher proportions of loss-to-follow-up, relative to younger adolescents (10–19 years of age) in the Western Cape. Noting that our study drew on a TB population in Cape Town, the largest city in the Western Cape, it corroborates that reported by Snow et al.

The corollary of what our data suggest to be an age-gradient in loss to follow-up, is that the factors that promote persistence in TB care during childhood may wane during adolescence and young adulthood. As children enter adolescence, they commonly, though not always, rebel against parental supervision, favour short term gain over long term benefit and often engage in activities that increase their socialization. They also seek employment and leave home, all of which make them vulnerable to being lost from care [29–32]. In their study on adolescents attending TB clinics in Botswana, Enane et al. did not distinguish younger adolescents (10–14 years old) from older adolescents (15–19 years old) [28]. Therefore, one reason for the discrepancy between their findings with our data and that of Snow et al., is that the grouping of younger adolescents with their older counterparts may have masked the age dependent risk of loss from TB care that occurs during adolescence. Alternatively, owing to the large variation in distribution of factors that hinder the wellbeing of young people across countries, loss to follow-up from TB care in Botswana need not occur via the same risk pathways as in Cape Town [4].

Retreatment TB emerged from the data as the largest independent predictor of loss to follow-up among young people in TB care. These findings imply that young people who became lost to follow-up may have had prior TB treatment, but either failed to complete it or had a new TB infection. In the former scenario young people could be failing to complete their treatment the first time round and returning to TB care after each failed episode, each carrying a higher chance of dropping out of TB care yet again. While the consequences of this for selection of DR-TB have not been investigated, they are possibly substantial.

We found that young men in Cape Town were more likely to become lost to follow-up from TB care than young women. Therefore, male gender may promote higher loss to follow-up in young people in Cape Town, as current evidence suggests that male individuals have reduced access to and engagement with TB care services in resource limited settings [33].

Our study showed that young women with TB bear a disproportionate burden of TB/HIV co-infection, as TB/HIV coinfection in young women in Cape Town exceeded 50%, well above the 27% coinfection prevalence reported among all young adult TB patients in the entire Western Cape [15]. As this finding reflects the entire cohort of young people treated for TB, with a 95–97% HIV testing result across age-groups, we can rule out selection bias as the source of this result. Hence the relatively higher burden of HIV prevalence among young women with TB is likely due to earlier sexually acquired HIV infection among young women relative to young men; a trend well established in sub-Saharan Africa [34], with a previous review identifying a peak in HIV infection among female TB patients in the 15–24 age group among Zambian and Kenyan cohorts [35].

Moreover, the higher burden of HIV infection among young women likely results in more female than male TB disease among young people. An earlier prospective study on risk factors for TB disease among adolescents (aged 12–18) in the Western Cape found female adolescents to have a 70% higher relative incidence rate of disease than male adolescents [13]. However, this association lacked statistical significance and HIV prevalence among incident cases was less than 1.0%. In light of our findings, prior HIV infection may drive markedly higher rates of TB in young women, which that study may have missed due to presumably low HIV risk in their population. Further research is necessary to understand how gender affects risk of TB disease among young people in resource limited settings.

A shortcoming of our analysis was that we could not ascertain the outcomes of all TB patients. In particular, patients who were reported as having transferred between health facilities, like those with outcome of “not evaluated” have no record of having either completed or discontinued TB care. This may have led to the systematic exclusion of patients at high risk for default, especially those who discontinue care after switching health facilities. However, as less than 3% of the cohort had the outcome of moving between health facilities during TB treatment and only 1.4% with outcome “not evaluated”, the impact thereof on our findings is not likely to have been substantial. The largest limitation of our study was the inability to ascertain data on occurrence and associations of DR-TB in this cohort, due to migration of all young people who develop DR-TB to a separate–and unlinked–database. This is a result of the current operational procedures of TB patient data collection in Cape Town, which similarly lacks linkage to HIV viral load data for patients with TB/HIV coinfection. Therefore, our study was unable to report on virological suppression for this patient population. Finally, our study lacks a much-needed assessment of the socio-political determinants of health and their role in TB treatment outcomes.

In the final analysis, our study showed a higher proportion of young women p with TB, driven by their disproportionate HIV prevalence relative to young men. We also identified increasing age, male gender, retreatment TB and TB/HIV co-infection as risk factors for increased loss to follow-up during TB treatment. The study highlights the need to focus on HIV prevention and retention in TB care among young people and reiterates the importance of addressing gender disparities in TB care [36]. We also emphasize the need for interventions that address TB/HIV co-infection, as young people co-infected with TB/HIV are particularly vulnerable to loss to follow-up. We support recommendations [15] for the introduction of adolescent tailored TB care that could improve patient retention in TB care, similar to improved outcomes reported by youth friendly HIV clinics.

## Supporting information

S1 Table Loss to follow-up among all adolescent and young adult TB patients across co-variates in multivariable logistic regression model. (DOCX)

S2 Table Loss to follow-up among HIV positive adolescent and young adult TB patients across co-variates in the multivariable logistic regression model. (DOCX)
