# Supplementary material for: HIV prevalence and determinants of loss-to-follow-up in adolescents and young adults with tuberculosis in Cape Town
Source: PLoS One. 2019 Feb 5;14(2):e0210937. doi: 10.1371/journal.pone.0210937 (PMC6363173; doi:10.1371/journal.pone.0210937)
Supplement: S1 Table — (DOCX) [file pone.0210937.s001.docx]

**S1 Table Loss-to-follow-up among all adolescent and young adult TB patients across co-variates in multivariable logistic regression model**

|  | % Lost-to-Follow Up (n) | Total N |
| --- | --- | --- |
| **Gender** |  |  |
| Female | 9.0% (1161) | 12961 |
| Male | 10.2% (1095) | 10776 |
| **HIV status** |  |  |
| HIV Negative | 8.0% (1352) | 16818 |
| HIV Positive  Unknown HIV status  **TB classification** | 13.3% (817)  11.1% (87) | 6137  782 |
| Pulmonary TB  EPTB  **Past history of TB**  New TB  Retreatment TB | 9.9% (2012)  7.3% (244)  7.4% (1515)  22.1% (741) | 20383  3354  20383  3354 |
