# Supplementary material for: HIV prevalence and determinants of loss-to-follow-up in adolescents and young adults with tuberculosis in Cape Town
Source: PLoS One. 2019 Feb 5;14(2):e0210937. doi: 10.1371/journal.pone.0210937 (PMC6363173; doi:10.1371/journal.pone.0210937)
Supplement: S2 Table — (DOCX) [file pone.0210937.s002.docx]

**S2 Table Loss-to-follow-up among HIV positive adolescent and young adult TB patients across co-variates in the multivariable logistic regression model**

|  | % Lost-to-Follow Up (n) | Total N |
| --- | --- | --- |
| **Age category** |  |  |
| 10-14 | 7.2% (27) | 373 |
| 15-19 | 13.2% (120) | 909 |
| 20-24 | 13.8 % (670) | 4855 |
| **Gender** |  |  |
| Female | 12.9% (617) | 4799 |
| Male | 14.9% (200) | 1138 |
| **ART status** |  |  |
| ART naïve at start of TB Rx | 13.5% (691) | 5107 |
| On ART at start of TB Rx | 12.3% (126) | 1027 |
| Unknown ART status | 0 | 3 |
| **CD4 counts** |  |  |
| <100 | 12.1% (181) | 1491 |
| 100-199 | 12.9% (169) | 1309 |
| 200-349 | 14.4% (213) | 1475 |
| 350-499 | 12.0% (107) | 890 |
| >=500 | 15.0% (105) | 701 |
| **TB classification** |  |  |
| Pulmonary  EPTB  **Past history of TB**  New TB  Retreatment TB | 13.7% (667)  12.0% (150)  11.2% (556)  22.1 % (261) | 4882  1255  4954  1183 |
